# Prevalence of Diagnosed and Undiagnosed HIV Infection - United States, 2008–2012

**Published:** 2015-06-26

**Authors:** H. Irene Hall, Qian An, Tian Tang, Ruiguang Song, Mi Chen, Timothy Green, Jian Kang

**Affiliations:** 1 Division of HIV/AIDS Prevention, National Center for HIV/AIDS, Viral Hepatitis, STD, and TB Prevention, CDC; 2 ICF International, Atlanta, Georgia; 3 Emory University, Atlanta, Georgia

Persons unaware of their human immunodeficiency virus (HIV) infection contribute nearly one third of ongoing transmission in the United States (1). Among the estimated 1.2 million persons living with HIV in the United States in 2011, 14% had undiagnosed infections (2). To accelerate progress toward reducing undiagnosed HIV infection, CDC and its partners have pursued an approach that includes expanding HIV testing in communities with high HIV infection rates (3). To measure the prevalence of diagnosed and undiagnosed HIV infection for the 50 states and the District of Columbia (DC), CDC analyzed data from the National HIV Surveillance System. In 42 jurisdictions with numerically stable estimates, HIV prevalence in 2012 ranged from 110 per 100,000 persons (Iowa) to 3,936 per 100,000 (DC). The percentage of persons living with diagnosed HIV ranged from 77% in Louisiana to ≥90% in Colorado, Connecticut, Delaware, Hawaii, and New York. In 39 jurisdictions with numerically stable estimates, the percentage of HIV cases with diagnosed infection among men who have sex with men (MSM) ranged from 75% in Louisiana to ≥90% in Hawaii and New York. These data demonstrate the need for interventions and public health strategies to reduce the prevalence of undiagnosed HIV infection. Because the percentage of persons with undiagnosed HIV varies by geographic area, efforts tailored to each area’s unique circumstances might be needed to increase the percentage of persons aware of their infection.

HIV surveillance data for persons aged ≥13 years from 50 states and DC reported to CDC through June 2014 were used to estimate the prevalence of diagnosed and undiagnosed HIV infection for 2008–2012. (Data for all years during the period 2008–2012 are available online at http://stacks.cdc.gov/view/cdc/31699.) Data were adjusted for reporting delays (2), missing transmission category (2), incorrect diagnosis dates, and underreporting. Although acquired immune deficiency syndrome (AIDS) has been reportable in all jurisdictions since the early 1980s, confidential name-based HIV reporting was implemented over time in different jurisdictions. To correct for erroneous HIV diagnosis dates resulting from the reporting of prevalent cases shortly after implementation of HIV reporting, the year of HIV diagnosis was adjusted among persons who received an AIDS diagnosis before and during the first 2 years after implementation of HIV reporting in a jurisdiction. AIDS cases were classified into two groups: 1) those diagnosed after 2 years of implementing HIV reporting (reference group) and 2) all other AIDS cases. In both groups, cases were stratified by year of AIDS diagnosis and vital status in December 2012. To ensure the same distribution of year of diagnosis in both groups, the distribution of year of HIV diagnosis in the reference group was used to adjust the year of HIV diagnosis of AIDS cases in the second group, by randomly distributing cases to earlier years in which the number of HIV diagnoses was less than expected and separately by jurisdiction of residence at AIDS diagnosis. Similarly, to adjust for underreporting of the number of HIV diagnoses before and during the first 2 years of implementation of HIV reporting, all HIV cases were classified into two groups: 1) HIV diagnoses after 2 years of implementing HIV reporting, or in jurisdictions with HIV reporting before 2000* (reference group) and 2) all other HIV cases. In both groups, cases were stratified by year of HIV diagnosis and AIDS status, both at diagnosis and at the end of study period.

The year of HIV diagnosis among cases of AIDS diagnosed during the same calendar year in the reference group was used to adjust the number of nonsimultaneous HIV and AIDS diagnoses (among persons with disease never classified as AIDS, to maintain the actual number ever classified as AIDS) in the second group of HIV cases so that the proportional distribution of same-year HIV and AIDS diagnosis was the same in both groups. This adjustment was done by jurisdiction of residence at HIV diagnosis. Individual adjustment weights were assigned to each case and combined with reporting delay weights for HIV diagnosis, AIDS diagnosis, and death, so annual numbers of HIV diagnoses, same-year AIDS diagnoses, and deaths could be obtained for any subpopulation.

Using the estimated annual number of HIV diagnoses and the severity of disease at diagnosis (i.e., whether the infection was classified as AIDS in the same calendar year the HIV diagnosis was made), a back-calculation model was fitted to estimate HIV prevalence, based on estimated cumulative HIV incidence (2). The overall HIV prevalence estimate was calculated by subtracting the estimated cumulative number of deaths that had occurred among those infected by the end of a given year from the estimated cumulative number of HIV infections. The estimated undiagnosed HIV prevalence was calculated by subtracting the estimated number of diagnosed HIV infections in living persons from the number of persons included in estimated overall HIV prevalence. Estimates for jurisdictions with an average of <60 diagnoses per year over the most recent 5 years (2008–2012) were considered numerically unstable.

In 42 jurisdictions with numerically stable estimates, the estimated prevalence of persons living with diagnosed or undiagnosed HIV infection in 2012 ranged from 110 per 100,000 persons (Iowa) to 3,936 per 100,000 persons (DC) (Table 1). The estimated percentage of persons living with HIV who had received a diagnosis of HIV by the end of 2012 ranged from 77% in Louisiana to ≥90% in Colorado, Connecticut, Delaware, Hawaii, and New York. During 2008–2012, HIV prevalence increased ≥5% in 36 jurisdictions, with numerically significant increases in 23 jurisdictions.† (An expanded table, presenting data for all years during the period 2008–2012, is available online at http://stacks.cdc.gov/view/cdc/31699.) The percentage of persons living with diagnosed HIV infection increased by ≥5% in eight jurisdictions (Arizona, DC, Iowa, Mississippi, Nebraska, New Mexico, North Carolina, and Rhode Island); however, these changes were not numerically significant.

In 39 jurisdictions with numerically stable estimates, the number of MSM living with HIV in 2012 ranged from 1,600 in Delaware and in Iowa to 134,400 in California (Table 2). The percentage of those who had their infection diagnosed ranged from 75% in Louisiana to ≥90% in Hawaii and New York.

## Discussion

The percentage of persons living with HIV who had received a diagnosis of HIV infection varied by jurisdiction. At the end of 2012, five jurisdictions (Colorado, Connecticut, Delaware, Hawaii, and New York) met the National HIV/AIDS Strategy objective to increase the percentage of persons living with HIV who know their serostatus to ≥90%, a critical component of the strategy to meet the goal of reducing new HIV infections in the Unites States (4). Among MSM, who constitute approximately 60% of persons diagnosed with HIV each year (2) and who are a target population for HIV testing, the estimated percentage with HIV who had received an HIV diagnosis was as low as 75% in Louisiana, with only two jurisdictions meeting the goal of ≥90%. Monitoring HIV prevalence can help in the planning for service needs. Increases in prevalence can indicate stable HIV incidence or increasing HIV incidence, with improved care and treatment prolonging survival; this is reflected in decreases in death rates among persons living with HIV during the same period (5). In jurisdictions where ≥90% of persons living with HIV had received an HIV diagnosis by the end of 2012, HIV prevalence was stable, which could indicate that the HIV spread has slowed.

HIV diagnosis is the essential first step in the HIV care continuum. Diagnosis allows persons to receive care and treatment to reduce viral load, increase immune function, and thereby reduce risk for transmission, morbidity, and mortality (6). Persons who are aware of their infection can also make behavioral changes to reduce transmission. CDC recommends that adolescents and adults be tested for HIV infection at least once and persons at increased risk for HIV infection (including MSM and persons who inject drugs) be tested at least annually (7). Decreases in undiagnosed HIV infection in recent years might be attributable to intensified testing efforts, and evidence suggests that the percentage of persons ever tested for HIV infection has increased (8) and that the time from infection to diagnosis has decreased (9).

The findings in this report are subject to at least two limitations. First, persons living with HIV might move from one jurisdiction to another, resulting in delays in updating residential information in surveillance data. Prevalence estimates were based on the most recent known address, so delays or errors in address reporting, or imbalanced in- and out-migration could affect jurisdictional estimates. Second, because HIV reporting was implemented over time by different jurisdictions, data adjustments were required to account for incomplete reporting and reporting of prevalent cases (delayed diagnosis years). The adjustments for incomplete reporting were conducted separately for high-morbidity jurisdictions and all other jurisdictions combined. This adjustment might not be accurate for low-morbidity jurisdictions, although results might appear stable.

**Summary**

What is already known on this topic?

Among the estimated 1.2 million persons living with human immunodeficiency virus (HIV) infection in the United States in 2011, 14% were living with undiagnosed infection. The majority of persons who received a diagnosis of HIV infection in 2011 were men who have sex with men (62%).

What is added by this report?

In 42 jurisdictions with numerically stable estimates, HIV prevalence in 2012 ranged from 110 per 100,000 persons (Iowa) to 3,936 per 100,000 (District of Columbia). The percentage of HIV-infected persons with diagnosed HIV ranged from 77% in Louisiana to ≥90% in Colorado, Connecticut, Delaware, Hawaii, and New York. Among men who have sex with men, the percentage of HIV cases that were diagnosed ranged from 75% in Louisiana to ≥90% in Hawaii and New York in 39 jurisdictions with numerically stable estimates.

What are the implications for public health practice?

To achieve the National HIV/AIDS Strategy’s objective to increase the percentage of persons living with HIV who know their serostatus to ≥90%, sustained efforts are needed to fully implement routine HIV testing. The percentage of persons with undiagnosed HIV varies by geographic area, and efforts tailored to each area’s unique needs and situations might be needed to increase the percentage of persons aware of their infection.

To advance the goals of the National HIV/AIDS Strategy (i.e., reducing new HIV infections, improving health outcomes among persons living with HIV, and reducing HIV-related disparities), CDC and its partners have been pursuing a prevention approach to maximize the impact of current HIV testing efforts (3). The results presented in this report show that although the overall percentage of persons living with HIV who have received a diagnosis of HIV infection is high, additional efforts are needed to ensure that all jurisdictions meet the goals of the strategy. Continued efforts to implement routine HIV screening in health care settings and focus on targeted testing in non-health care settings to access populations in communities with disproportionately high HIV burden, including the 10 jurisdictions with the highest number of undiagnosed infections§ comprising about 68% of all undiagnosed infections, might help further reduce undiagnosed HIV infection. With an estimated 40% of persons living with HIV engaged in HIV medical care, 37% prescribed antiretroviral therapy (ART), and 30% having achieved viral suppression in 2011, improvements are also critical in other steps of the continuum of care to reach the United Nations’ goals of ≥90% of persons living with diagnosed HIV infection receiving ART and ≥90% of persons receiving ART having viral suppression by 2020, and ultimately reduce HIV transmission in the United States (6,10).

## Figures and Tables

**TABLE 1:** Estimated* number of persons aged ≥13 years with HIV infection (diagnosed and undiagnosed), and percentage of those with diagnosed HIV infection, by jurisdiction† — United States, 2012

	Persons living with diagnosed or undiagnosed HIV infection				Persons living with undiagnosed HIV infection		Persons living with diagnosed HIV infection	
			
Jurisdiction	No.	(95% CI)	Rate§	(95% CI)	No.	(95% CI)	%	(95% CI)
Alabama	14,400	(13,600–15,300)	358	(338–381)	2,300	(1,500–3,200)	84.0	(78.6–89.2)
Alaska¶	790	(710–900)	133	(120–152)	70	(0–190)	91.1	(78.0–99.9)
Arizona	16,200	(15,700–16,700)	301	(292–310)	1,900	(1,400–2,500)	88.3	(85.0–91.4)
Arkansas	5,800	(5,500–6,200)	238	(226–254)	1,000	(620–1,400)	82.8	(77.2–89.3)
California	183,300	(180,100–186,900)	583	(573–595)	20,700	(17,100–24,300)	88.7	(86.7–90.3)
Colorado	12,600	(12,100–13,100)	294	(282–305)	1,300	(740–1,800)	89.7	(86.1–93.3)
Connecticut	13,500	(12,900–14,100)	444	(424–464)	1,300	(850–1,800)	90.4	(86.8–93.9)
Delaware	4,300	(4,000–4,500)	559	(520–585)	430	(120–720)	90.0	(83.5–96.9)
District of Columbia	21,700	(20,900–22,400)	3,936	(3,791–4,063)	2,300	(1,400–3,100)	89.4	(86.2–93.2)
Florida	127,900	(125,400–130,000)	777	(761–789)	15,900	(13,500–17,900)	87.6	(86.1–89.3)
Georgia	57,300	(55,700–58,700)	706	(686–723)	10,700	(9,000–12,300)	81.3	(79.1–83.8)
Hawaii	3,500	(3,300–3,700)	300	(283–318)	250	(0–500)	92.9	(86.3–100.0)
Idaho¶	1,100	(1,000–1,200)	86	(78–93)	100	(0–220)	90.9	(81.5–100.0)
Illinois	45,700	(44,100–47,000)	427	(413–440)	7,500	(5,800–8,700)	83.6	(81.3–86.9)
Indiana	11,400	(10,700–11,900)	211	(198–220)	1,700	(970–2,200)	85.1	(80.7–90.0)
Iowa	2,800	(2,600–3,000)	110	(102–117)	520	(280–750)	81.4	(74.8–89.2)
Kansas	3,700	(3,400–3,900)	157	(144–165)	560	(310–780)	84.9	(78.8–91.0)
Kentucky	8,300	(7,900–8,700)	228	(217–239)	1,200	(780–1,700)	85.5	(80.7–90.6)
Louisiana	22,600	(21,700–23,500)	596	(572–619)	5,100	(4,200–6,000)	77.4	(74.3–80.5)
Maine¶	1,800	(1,600–1,900)	157	(140–166)	90	(0–230)	95.0	(86.8–100.0)
Maryland	43,300	(41,500–45,000)	880	(843–914)	8,100	(6,200–9,900)	81.3	(77.8–85.0)
Massachusetts	27,000	(26,200–27,900)	477	(463–493)	4,100	(3,300–5,000)	84.8	(81.6–87.5)
Michigan	17,500	(16,800–18,200)	211	(203–219)	2,700	(1,900–3,500)	84.6	(80.5–88.1)
Minnesota	8,400	(8,000–8,800)	188	(180–197)	1,200	(760–1,600)	85.7	(81.2–90.0)
Mississippi	10,300	(9,600–10,900)	420	(392–445)	1,700	(1,100–2,200)	83.5	(79.3–88.1)
Missouri	13,200	(12,600–13,900)	263	(251–277)	1,800	(1,300–2,600)	86.4	(81.6–90.1)
Montana¶	650	(550–730)	77	(65–86)	30	(0–130)	95.4	(80.7–99.7)
Nebraska	2,200	(2,000–2,400)	145	(132–158)	290	(110–490)	86.8	(79.4–94.4)
Nevada	9,600	(9,100–10,100)	421	(399–443)	1,400	(740–1,900)	85.4	(81.0–91.4)
New Hampshire¶	1,600	(1,500–1,800)	141	(132–159)	120	(0–310)	92.5	(82.4–100.0)
New Jersey	43,100	(41,800–44,500)	580	(563–599)	6,800	(5,500–8,200)	84.2	(81.3–87.0)
New Mexico	3,600	(3,400–3,800)	210	(199–222)	400	(160–630)	88.9	(82.7–95.0)
New York	177,000	(174,800–179,600)	1,070	(1,057–1,086)	12,600	(10,000–15,400)	92.9	(91.4–94.3)
North Carolina	32,000	(31,100–32,900)	395	(384–406)	4,200	(3,100–5,200)	86.9	(84.1–89.9)
North Dakota¶	330	(270–390)	56	(46–67)	20	(0–100)	93.9	(73.9–100.0)
Ohio	22,900	(22,100–23,700)	237	(229–245)	4,200	(3,400–5,000)	81.7	(78.7–84.7)
Oklahoma	6,700	(6,300–7,100)	214	(201–227)	1,100	(680–1,600)	83.6	(78.4–89.5)
Oregon	8,400	(7,900–8,700)	256	(241–265)	1,100	(540–1,500)	86.9	(82.1–92.3)
Pennsylvania	40,900	(39,700–42,100)	378	(367–389)	5,700	(4,500–6,700)	86.1	(83.8–88.8)
Rhode Island	2,500	(2,300–2,700)	278	(256–300)	280	(10–490)	88.8	(81.1–98.9)
South Carolina	19,300	(18,200–20,100)	489	(461–510)	3,200	(2,000–4,000)	83.4	(79.2–88.3)
South Dakota¶	520	(450–590)	76	(66–86)	90	(10–180)	82.7	(68.7–98.3)
Tennessee	19,200	(18,300–19,800)	357	(340–368)	2,700	(1,700–3,400)	85.9	(82.4–89.9)
Texas	104,300	(101,800–106,200)	497	(485–506)	18,000	(15,300–19,800)	82.7	(81.2–84.7)
Utah	2,900	(2,700–3,200)	132	(123–146)	430	(160–700)	85.2	(76.6–94.1)
Vermont¶	810	(730–890)	150	(135–165)	0	(0–50)	100.0	(93.7–100.0)
Virginia	25,100	(24,200–25,900)	367	(354–379)	3,200	(2,300–4,100)	87.3	(83.9–90.4)
Washington	15,400	(14,700–16,200)	268	(256–282)	1,900	(1,200–2,600)	87.7	(83.7–91.5)
West Virginia	2,200	(2,000–2,400)	139	(126–152)	330	(150–520)	85.0	(76.6–92.6)
Wisconsin	6,400	(6,000–6,900)	134	(125–144)	980	(450–1,530)	84.7	(77.7–92.4)
Wyoming¶	320	(260–390)	67	(55–82)	40	(0–110)	87.5	(68.6–100.0)
**Total** **	**1,218,400**	**(1,207,100–1,228,200)**	**467**	**(462.5–470.5)**	**156,300**	**(144,100–165,900)**	**87.2**	**(86.4–88.0)**

**Abbreviations:** HIV = human immunodeficiency virus; CI = confidence interval.

* Estimates were derived by using back-calculation. Estimates were rounded to the nearest 100 for numbers >1,000 and to the nearest 10 for numbers <1,000 to reflect the uncertainty inherent in statistical estimates.

† Persons whose most recent known address or residence at death is in the jurisdiction by December 31, 2012.

§ Per 100,000 population.

¶ Estimates for jurisdictions with <60 diagnoses per year (average) over the most recent 5 years (2008–2012) are considered numerically unstable.

** Because column totals were calculated independently and to correspond to methods for national estimates with 24-month reporting delay, the values in each column might not sum to the column total.

**TABLE 2:** Estimated* number of males aged ≥13 years with HIV infection (diagnosed and undiagnosed) attributed to male-to-male sexual contact and percentages of those with diagnosed HIV infection, by jurisdiction† —United States, 2012

	Persons living with diagnosed or undiagnosed HIV infection		Persons living with undiagnosed HIV infection		Persons living with diagnosed HIV infection	
			
Jurisdiction	No.	(95% CI)	No.	(95% CI)	%	(95% CI)
Alabama	7,900	(7,400–8,400)	1,600	(990–2,000)	79.7	(75.5–85.3)
Alaska§	410	(350–480)	20	(0–270)	95.1	(77.4–96.7)
Arizona	10,500	(10,100–11,000)	1,200	(630–1,800)	88.6	(83.9–93.4)
Arkansas	3,500	(3,200–3,900)	800	(450–1,260)	77.1	(69.0–83.8)
California	134,400	(132,700–136,400)	16,400	(14,100–18,500)	87.8	(86.4–89.2)
Colorado	8,900	(8,500–9,200)	950	(510–1,360)	89.3	(85.1–93.8)
Connecticut	4,600	(4,300–4,900)	710	(320–1,000)	84.6	(78.8–92.1)
Delaware	1,600	(1,500–1,800)	240	(10–430)	85.0	(75.9–96.6)
District of Columbia	11,300	(10,900–11,900)	1,400	(820–2,000)	87.6	(82.5–91.9)
Florida	60,500	(58,900–62,000)	8,100	(6,500–9,600)	86.6	(84.1–88.9)
Georgia	33,100	(31,800–34,100)	6,900	(5,400–8,000)	79.2	(76.3–82.9)
Hawaii	2,500	(2,400–2,700)	220	(0–640)	91.2	(83.1–95.7)
Idaho§	630	(560–710)	80	(0–220)	87.3	(72.1–96.9)
Illinois	27,800	(26,600–28,600)	5,300	(4,200–6,200)	80.9	(78.3–84.3)
Indiana	6,900	(6,500–7,300)	1,000	(530–1,420)	85.5	(80.3–91.8)
Iowa	1,600	(1,400–1,800)	330	(130–550)	79.4	(69.5–89.3)
Kansas	2,200	(2,000–2,400)	380	(170–590)	82.7	(75.8–90.3)
Kentucky	5,300	(5,000–5,600)	890	(480–1,210)	83.2	(77.8–90.5)
Louisiana	10,700	(10,000–11,300)	2,700	(2,000–3,300)	74.8	(70.0–79.9)
Maine§	1,200	(1,000–1,300)	90	(0–460)	92.5	(83.7–94.9)
Maryland	16,200	(15,300–16,900)	3,900	(2,900–4,900)	75.9	(71.7–80.5)
Massachusetts	12,200	(11,500–12,800)	2,000	(1,300–2,700)	83.6	(79.0–87.9)
Michigan	10,900	(10,100–11,600)	1,900	(1,200–2,700)	82.6	(76.8–88.1)
Minnesota	5,200	(5,000–5,500)	770	(360–1,200)	85.2	(78.6–91.9)
Mississippi	5,400	(5,000–5,900)	1,200	(740–1,700)	77.8	(70.5–84.7)
Missouri	9,100	(8,600–9,500)	1,500	(960–1,900)	83.5	(78.7–88.7)
Montana§	420	(360–480)	30	(0–220)	92.9	(75.9–95.1)
Nebraska§	1,300	(1,200–1,400)	190	(40–320)	85.4	(76.9–95.4)
Nevada	6,500	(6,100–6,800)	1,000	(590–1,400)	84.6	(79.3–90.3)
New Hampshire§	950	(830–1,050)	120	(0–290)	87.4	(77.6–94.7)
New Jersey	16,800	(15,800–17,800)	3,700	(2,400–4,800)	78.0	(73.5–84.5)
New Mexico	2,400	(2,200–2,600)	280	(50–480)	88.3	(81.0–97.7)
New York	75,900	(73,900–78,200)	7,700	(5,700–10,000)	89.9	(87.0–92.4)
North Carolina	16,100	(15,400–16,600)	2,600	(1,900–3,400)	83.9	(80.0–87.3)
North Dakota§	190	(130–230)	20	(0–150)	89.5	(59.2–95.4)
Ohio	14,800	(14,200–15,400)	3,100	(2,300–3,800)	79.1	(75.1–83.0)
Oklahoma	4,100	(3,800–4,400)	740	(370–1,060)	82.0	(75.9–89.5)
Oregon	5,800	(5,500–6,200)	850	(350–1,230)	85.3	(79.4–92.8)
Pennsylvania	16,100	(15,200–17,000)	2,700	(1,800–3,600)	83.2	(78.3–87.8)
Rhode Island§	1,100	(1,000–1,300)	200	(50–350)	81.8	(71.6–92.3)
South Carolina	9,500	(8,900–10,000)	2,000	(1,400–2,600)	78.9	(73.5–85.0)
South Dakota§	200	(160–240)	30	(0–80)	85.0	(66.5–97.7)
Tennessee	11,000	(10,600–11,500)	1,800	(1,300–2,200)	83.6	(80.1–87.6)
Texas	62,400	(61,000–63,700)	12,100	(10,400–13,200)	80.6	(78.7–83.0)
Utah	1,700	(1,500–1,800)	250	(40–440)	85.3	(75.1–95.5)
Vermont§	520	(450–590)	0	(0–30)	100.0	(94.5–100.0)
Virginia	13,500	(12,900–14,200)	2,000	(1,300–2,700)	85.2	(80.6–89.4)
Washington	10,400	(9,900–10,800)	1,300	(650–1,700)	87.5	(83.2–93.1)
West Virginia§	1,200	(1,100–1,300)	200	(40–350)	83.3	(73.3–92.1)
Wisconsin	4,000	(3,700–4,200)	650	(320–980)	83.8	(77.2–89.9)
Wyoming§	180	(140–220)	40	(0–120)	77.8	(57.6–94.2)
**Total** ¶	**666,900**	**(659,900–674,300)**	**98,700**	**(91,200–105,400)**	**85.2**	**(84.2–86.2)**

**Abbreviations:** HIV = human immunodeficiency virus; CI = confidence interval.

* Estimates were derived by using back-calculation. Estimates were rounded to the nearest 100 for numbers >1,000 and to the nearest 10 for numbers <1,000 to reflect the uncertainty inherent in statistical estimates.

† Persons whose most recent known address or residence at death is in the jurisdiction by December 31, 2012.

§ Estimates for jurisdictions with <60 diagnoses per year (average) over the most recent 5 years (2008–2012) are considered numerically unstable.

¶ Because column totals were calculated independently and to correspond to methods for national estimates with 24-month reporting delay, the values in each column might not sum to the column total.

## Footnotes

* Except Texas and Florida, which reported few HIV cases diagnosed before 1999 and 1997, respectively.

† Alabama, Arizona, Arkansas, California, District of Columbia, Florida, Georgia, Illinois, Kentucky, Louisiana, Maryland, Massachusetts, Michigan, Minnesota, Missouri, New Jersey, North Carolina, Ohio, Oklahoma, Pennsylvania, Tennessee, Texas, and Virginia.

§ California, Florida, Georgia, Illinois, Louisiana, Maryland, New Jersey, New York, Ohio, and Texas.
